# Feasibility of one-month home-based HRV monitoring in ASD: a case study using smart clothing technology

**DOI:** 10.3389/fdgth.2025.1741400

**Published:** 2026-01-09

**Authors:** Soichiro Matsuda, Yurina Shinohara

**Affiliations:** 1 Behavioral Design Laboratory, Institute of Human Sciences, University of Tsukuba, Tsukuba, Japan; 2 Behavioral Design Laboratory, College of Psychology, School of Human Sciences, University of Tsukuba, Tsukuba, Japan

**Keywords:** autism spectrum disorder (ASD), digital mental health, heart rate variability (HRV), home-based monitoring, personalized care, smart clothing, wearable sensing

## Abstract

**Background:**

Sleep disturbances and autonomic dysregulation are common in autism spectrum disorder (ASD), yet few studies have examined long-term nocturnal heart rate variability (HRV) in home settings.

**Objective:**

This study evaluated the feasibility of one-month home-based HRV monitoring using smart clothing in a preschooler with ASD, and explored whether nocturnal HRV predicts next-day problem behaviors.

**Methods:**

HRV was recorded nightly for 25 valid days using a garment-type wearable ECG. Problem behaviors were reported daily by caregivers. HRV indices were compared between nights preceding days with and without problem behaviors using Wilcoxon signed-rank tests.

**Results:**

No significant differences in total sleep time or HRV indices were found between the two day types.

**Conclusion:**

Although HRV did not predict next-day behavior, the study demonstrates the feasibility and methodological transparency of long-term home-based physiological monitoring in young children with ASD.

## 1 Introduction

Autism spectrum disorder (ASD) involves difficulties in social communication and restricted, repetitive behaviors (1). Over 90% of children with ASD exhibit at least one type of problem behavior, such as self-injury or tantrums (2, 3). Furthermore, children with ASD are more likely to have comorbid physical and mental health conditions, including epilepsy and depression (4). Among these, sleep disturbances are some of the most frequently reported comorbidities, with an estimated 50%–80% of children with ASD experiencing sleep-related issues (5, 48). Compared to typically developing (TD) children, those with ASD exhibit a higher prevalence of difficulties such as sleep-onset delay, nighttime awakenings, and suboptimal sleep hygiene, and these symptoms often persist chronically (6, 49).

Research has also demonstrated associations between sleep problems and core ASD symptoms. For instance, children with ASD who obtain less total sleep time per night tend to exhibit greater symptom severity, particularly in the domain of social communication deficits (7). In addition, shorter sleep duration and frequent nighttime awakenings have been shown to predict the presence of stereotyped behaviors (8). Heightened sensory hypersensitivity is also linked to more severe sleep difficulties in children with ASD. Specifically, greater sensory sensitivity has been associated with increased sleep-onset delays, more frequent nighttime awakenings, and reduced total sleep duration (9, 10).

Numerous studies have reported atypical autonomic nervous system (ANS) functioning in individuals with ASD (11, 12). A commonly used index of ANS activity is heart rate variability (HRV), which refers to the variation in intervals between successive R-waves (RR intervals) on an electrocardiogram. HRV can be measured noninvasively, making it a widely adopted parameter in recent psychophysiological research [e.g., (13, 14)]. Studies have shown that children with ASD exhibit lower resting-state HRV than their TD peers, and this reduction in HRV has been associated with atypical attentional responses, difficulties in emotion regulation, and deficits in social communication (15, 16). However, recent meta-analytic and theoretical work suggests that autonomic differences in ASD and other neurodevelopmental conditions may be more heterogeneous than previously assumed, and not all studies report reduced HRV in ASD (17, 18). This variability underscores the importance of transparent and replicable protocols that can support nuanced interpretation of HRV findings.

Given that ANS activity fluctuates cyclically during sleep, HRV is also widely used as a physiological indicator of sleep state and quality (19, 20). Under typical conditions, sympathetic nervous system activity increases during rapid eye movement (REM) sleep—resulting in elevated heart rate—while parasympathetic activity dominates during non-REM (NREM) sleep—resulting in a lower heart rate (20). In contrast, children with ASD tend to show elevated sympathetic activity and higher heart rates during REM sleep, as well as reduced parasympathetic activity and persistently elevated heart rates during NREM sleep, leading to lower HRV across the night compared to TD children (21). However, to date, no studies have investigated HRV during sleep specifically in preschool-aged children with ASD.

Sleep plays an important role in emotion regulation and daytime functioning, and in autistic children poorer sleep quality has been associated with more behavior problems and social difficulties (7). From this perspective, nocturnal HRV may index the extent to which autonomic functioning “resets” during sleep, which may subsequently influence behavioral and emotional regulation the following day. Examining night-to-day carryover effects therefore provides a mechanistic rationale for focusing on nocturnal HRV rather than solely on daytime physiological indices.

Although some studies have examined the relationship between sleep problems and daytime behavioral problems, or assessed daytime resting-state HRV in children with ASD, the association between nocturnal HRV and subsequent problem behaviors remains largely unexplored. Although several studies have measured HRV during sleep, most have relied on polysomnography [e.g.m (13, 19, 21, 22)]. Polysomnography offers the advantage of accurately capturing a wide range of physiological signals, including electrocardiography (ECG), electroencephalography (EEG), and eye movements during sleep (23, 24). However, it requires the attachment of multiple electrodes to the body, which cannot be easily removed and may be particularly distressing for infants and young children—especially those with sensory hypersensitivity, as is common in ASD (24). Moreover, polysomnography is expensive and requires specialized expertise, often necessitating visits to laboratories or hospitals. This makes it less suitable for naturalistic, home-based monitoring (25). In response to these limitations, recent attention has shifted to wearable garment-type devices (e.g., 25–27). These devices use conductive textiles to measure HRV with minimal tactile stimulation, enabling comfortable and unobtrusive data collection during both active and resting states (26). They are better tolerated by individuals with sensory sensitivities, including children with ASD, and have been found suitable for use in this population (28). Additionally, unlike traditional polysomnography, these wireless, portable devices can be used at home, making them ideal for continuous or long-term monitoring in natural environments.

Building on this perspective, the present study aims to longitudinally and quantitatively evaluate the everyday sleep patterns of a child with ASD by measuring electrocardiographic data over a one-month period using a wearable garment-type device. Specifically, we investigate whether variations in HRV during sleep predict subsequent daytime problem behaviors. We hypothesize that lower HRV and increased sympathetic dominance during sleep will be associated with a higher likelihood of problem behaviors occurring the following day.

## 2 Method

### 2.1 Participant

Two preschool-aged boys with autism spectrum disorder (ASD), aged 4 years and 5 months and 4 years and 8 months respectively, were recruited from clients of the Educational Counseling Center at the University of Tsukuba. However, the child aged 4 years and 5 months exhibited severe sensory hypersensitivity that made it difficult for him to tolerate the wearable device, and therefore he could not continue participation in the study. Consequently, the final participant was a single boy aged 4 years and 8 months.

To assess the severity of the participant's ASD symptoms, we used the Social Responsiveness Scale, Second Edition [SRS-2 (29, 30)], which evaluates impairments in social behavior in individuals with ASD. The questionnaire was completed by the participant's caregiver and includes 65 items rated on a 4-point Likert scale: “not true,” “sometimes true,” “often true,” or “almost always true.” The SRS-2 provides raw scores and T-scores for five treatment subscales: Social Awareness, Social Cognition, Social Communication, Social Motivation, and Restricted Interests and Repetitive Behavior. Higher total scores and T-scores indicate greater severity of impairment. The measure also yields two DSM-5–compatible subscales: Social Communication and Interaction (SCI) and Restricted Interests and Repetitive Behavior (RRB). The participant's total raw score was 93, with a T-score of 85, which falls in the range indicating severe difficulties in social interaction.

In addition, to evaluate the participant's overall emotional and behavioral problems, we administered the Japanese version of the Child Behavior Checklist for ages 4–18 [CBCL/4–18 (31, 32)]. The caregiver was asked to rate each item based on the extent to which the child exhibited various problem behaviors, using a 3-point scale: “very true or often true,” “somewhat or sometimes true,” or “not true.” The CBCL includes eight syndrome scales: Withdrawn, Somatic Complaints, Anxious/Depressed, Social Problems, Thought Problems, Attention Problems, Delinquent Behavior, and Aggressive Behavior, which are grouped into higher-order scales: Internalizing Problems and Externalizing Problems, along with a Total Problems score. The participant's total CBCL score was 44, with an internalizing score of 5 and an externalizing score of 9. The total score was within the clinical range, while the internalizing and externalizing scores fell within the normal range.

This study was approved by the Research Ethics Committee of the Faculty of Human Sciences, University of Tsukuba (Approval No. 2020-164 A). Prior to participation, the caregiver was provided with a verbal explanation by a first author regarding the study's objectives and procedures. The explanation emphasized that participation was voluntary, that consent could be withdrawn at any time without penalty, and that refusal to participate would not result in any disadvantage.

### 2.2 Apparatus

**Figure 1:**
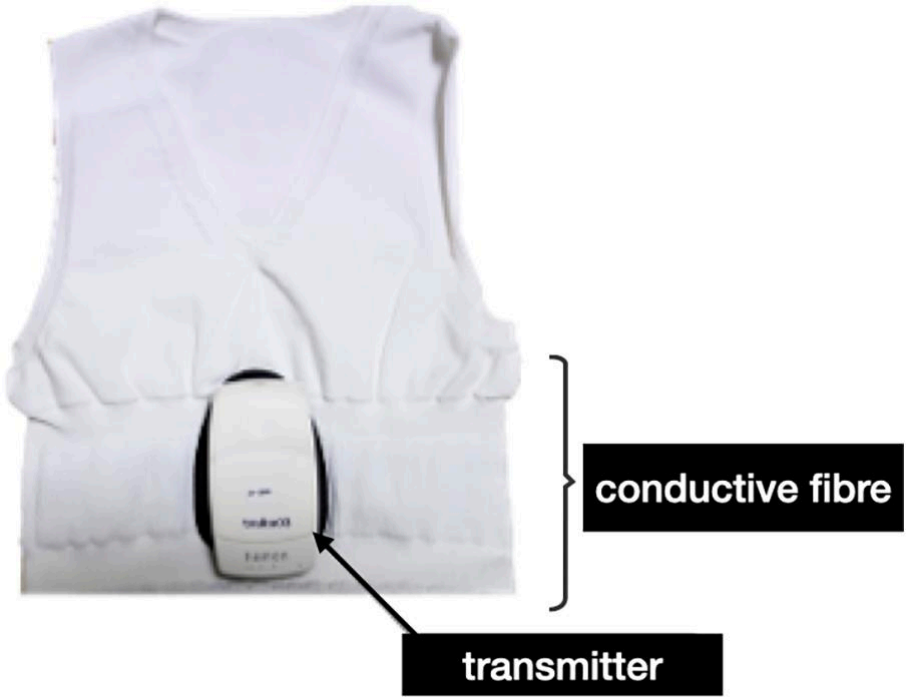
Smart clothing device (hamon®) used for nocturnal ECG monitoring. The garment incorporates silver-plated conductive textile fibers (AGposs®) knitted into the chest area, allowing dry-electrode ECG acquisition without gels or adhesive patches. A small transmitter module attaches to a snap connector located at the chest and wirelessly transmits ECG data to a smartphone and cloud platform. The garment is worn directly against the skin like a standard undershirt, minimizing tactile load and enabling unobtrusive overnight monitoring for preschool children with ASD.

A wearable undershirt-type device using conductive fabric, hamon®, developed by Mitsufuji Corporation, was used for physiological data collection (Figure 1). The hamon® system consists of a transmitter embedded in the garment that collects biosignals and transmits them via Bluetooth to a smartphone for self-monitoring. The garment uses silver-plated conductive textile fibers (AGposs®) integrated into the chest area, enabling dry-electrode ECG acquisition without gels or adhesive patches. The data are then stored in the Mitsufuji cloud system and subsequently transmitted to the principal investigator through the cloud platform. Electrocardiographic signals were sampled at a frequency of 250 Hz, with a data acquisition interval of 4 ms. Prior to measurement, the participant's chest circumference, epigastric circumference, waist circumference, back length, and shoulder width were measured to create a custom-fitted smart garment tailored to the child's body dimensions.

### 2.3 Procedure

Prior to the start of physiological data collection, the participant's caregiver was asked to complete two standardized questionnaires: the Social Responsiveness Scale, Second Edition (SRS-2) to assess ASD symptom severity, and the Japanese version of the Child Behavior Checklist (CBCL) to assess behavioral problems. The participant was instructed to wear the hamon® smart garment each night during sleep over a one-month period at home. Throughout the data collection period, the caregiver was also asked to record the participant's daily wake and sleep times, as well as the presence or absence of problem behaviors each day using a Google Form. The reporting form was sent automatically each night at 20:00, a schedule determined collaboratively with the caregiver prior to the study. The form presented a set of items adapted from the Japanese version of the Aberrant Behavior Checklist [ABC (33, 34)], and the caregiver was asked to indicate whether any of these behaviors had occurred during the day. These items were not individualized for this participant; rather, they were provided as a standard list to support daily reporting. If none of the listed items applied, the caregiver could describe any other observed challenging behavior using an open-ended field. A day was classified as a “problem behavior day” if at least one listed item or any free-text behavior was endorsed. The caregiver remained blind to HRV data throughout the study.

### 2.4 Data analysis

Electrocardiographic data obtained from the hamon® device were analyzed using Python (version 3.8) with an open-source package: *NeuroKit2* [version 0.1.4.1 (35);] and *hrv-analysis* (version 1.0.4). From these data, heart rate variability (HRV) indices were computed. To examine whether there were differences in total sleep duration and HRV indices between nights preceding days with reported problem behaviors and those without, Wilcoxon signed-rank tests were conducted. Given the single-case design, the small number of nights with problem behaviors (*n* = 5), and the non-normal distribution of several HRV indices, Wilcoxon signed-rank tests were selected. These analyses were treated as exploratory and intended to illustrate the analytic approach rather than provide definitive inferential conclusions. ECG data were collected on 32 nights in total. However, five nights had no corresponding ECG file due to device non-wear or technical recording failures, and one night (the final recording night) did not have a corresponding next-day behavior report. In addition, one recording was truncated at approximately 4:00 a.m., well before the child's typical wake time, and therefore did not sufficiently capture the full sleep period. These seven nights were excluded as invalid, leaving 25 nights for the final analysis.

### 2.5 Heart rate variability measures

Heart rate variability (HRV) parameters are commonly categorized into time-domain and frequency-domain measures. The time-domain parameters calculated in this study are listed in Table 1, and the frequency-domain parameters are shown in Table 2. Time-domain parameters are derived from the RR intervals, which represent the time intervals between successive R-waves—the highest peaks in the electrocardiogram waveform. In contrast, frequency-domain parameters are obtained by performing a Fourier transform on the RR interval data to compute the power spectral density (36). Among the time-domain indices, the root mean square of successive differences (rMSSD) and the percentage of successive RR intervals that differ by more than 50 ms (pNN50) are considered indicators of parasympathetic nervous system activity; higher values reflect greater parasympathetic dominance (19). In the frequency domain, the ratio of low-frequency to high-frequency power (LF/HF) is used as an index of autonomic balance, with higher values indicating increased sympathetic activity and lower values suggesting greater parasympathetic activity (20).

**Table 1:** Time-domain heart rate variability (HRV) parameters.

Parameter	Unit	Description
AVNN	ms	Mean of all RR intervals over a 5-minute window.
SDNN	ms	Standard deviation of RR intervals; reflects overall HRV.
rMSSD	ms	Root mean square of successive differences between adjacent RR intervals; reflects parasympathetic activity.
pNN50	%	Percentage of adjacent RR intervals differing by more than 50 ms; an index of parasympathetic tone.
HR	bpm	Mean heart rate per minute.

**Table 2:** Frequency-Domain heart rate variability (HRV) parameters.

Parameter	Description
LF (Low Frequency)	Power in the low-frequency band (0.04–0.15 Hz); reflects both sympathetic and parasympathetic activity.
HF (High Frequency)	Power in the high-frequency band (0.15–0.40 Hz); reflects parasympathetic activity.
Normalized LF	Normalized index of LF power; reflects sympathetic activity.
Normalized HF	Normalized index of HF power; reflects parasympathetic activity.
LF/HF Ratio	Ratio of LF to HF power; reflects the balance between sympathetic and parasympathetic nervous system activity.

## 3 Results

### 3.1 Reports of problem behaviors

Among the 25 days included in the final analysis, problem behaviors were reported on 5 days. The reported behaviors included “biting,” “hypersensitive responses to sounds,” and “inappropriate defecation (i.e., failure to use the toilet).”

### 3.2 Total sleep duration and HRV measures

For total sleep duration and each HRV parameter, means and standard deviations were calculated across (1) all 25 days, (2) the 5 nights preceding days with problem behaviors, and (3) the 20 nights preceding days without problem behaviors (see Table 3).

**Table 3:** Means, standard deviations, and Wilcoxon signed-rank test results for sleep duration and HRV parameters*.*

Variable	Overall Mean (*SD*)	Preceding Problem Behavior Day Mean (*SD*)	Preceding Non-Problem Day Mean (*SD*)	*p*-value
Total Sleep Time (min)	566.61 (31.76)	585.00 (19.36)	563.07 (32.71)	.34
AVNN (ms)	818.82 (30.91)	822.03 (42.48)	818.02 (28.70)	.59
SDNN (ms)	103.67 (18.25)	89.71 (28.38)	107.16 (13.68)	.79
rMSSD (ms)	89.10 (16.69)	92.06 (19.34)	88.36 (16.44)	.42
pNN50 (%)	42.24 (7.53)	39.91 (10.34)	42.82 (6.89)	.59
Mean HR (bpm)	74.84 (2.85)	74.46 (3.90)	74.94 (2.65)	.59
LF (ms^2^)	1,732.39 (458.97)	1,781.58 (546.71)	1,720.09 (449.02)	.79
HF (ms^2^)	1,391.20 (478.32)	1,386.44 (518.89)	1,392.39 (482.80)	.59
Normalized LF (%)	56.06 (4.13)	56.82 (3.83)	55.88 (4.27)	.59
Normalized HF (%)	43.94 (4.13)	43.18 (3.83)	44.12 (4.27)	.59
LF/HF Ratio	1.30 (0.24)	1.33 (0.23)	1.29 (0.25)	.59

HRV, heart rate variability; HR, heart rate; LF, clow-frequency power; HF, high-frequency power; AVNN, average of NN intervals; SDNN, standard deviation of NN intervals; rMSSD, root mean square of successive differences; pNN50, percentage of successive NN intervals differing by more than 50 ms. All *p*-values were derived from Wilcoxon signed-rank tests comparing nights preceding problem behavior days and non-problem behavior days.

### 3.3 Comparison between nights preceding problem behavior and Non-problem behavior days

To examine whether sleep characteristics differed depending on the presence or absence of problem behaviors on the following day, Wilcoxon signed-rank tests were conducted to compare the nights preceding behavior problem days (*n* = 5) with those preceding non-problem days (*n* = 20). The results revealed no statistically significant differences in any of the variables examined.

Descriptively, nights preceding problem behavior days showed slightly shorter total sleep time and marginally lower SDNN, whereas most HRV indices were otherwise similar across conditions. However, noticeable night-to-night variability was observed in several HRV measures (e.g., SDNN, rMSSD, pNN50), even on nights with comparable caregiver-reported sleep duration. These fluctuations ranged by approximately 50–70 ms for SDNN and rMSSD and by more than 20 percentage points for pNN50. In contrast, mean heart rate showed relatively minimal variability across nights. No extreme outlier nights disproportionately influenced the group means.

Specifically, total sleep duration did not differ between the two conditions (*V* = 11.3, *p* = .34). Likewise, time-domain HRV indices such as the average of NN intervals (AVNN; *V* = 10, *p* = .59), the standard deviation of NN intervals (SDNN; *V* = 6, *p* = .79), the root mean square of successive differences (rMSSD; *V* = 11, *p* = .42), and the percentage of NN intervals differing by more than 50 ms (pNN50; *V* = 5, *p* = .59) all showed no significant differences. Additionally, the mean heart rate (*V* = 5, *p* = .59) and frequency-domain parameters—including low-frequency power (LF; *V* = 9, *p* = .79), high-frequency power (HF; *V* = 10, *p* = .59), normalized LF (*V* = 5, *p* = .59), normalized HF (*V* = 10, *p* = .59), and the LF/HF ratio (*V* = 5, *p* = .59)—also did not significantly differ between the two types of nights.

## 4 Discussion

The primary aim of this study was to evaluate the feasibility of one-month home-based nocturnal HRV monitoring using smart clothing in a preschooler with ASD. A secondary exploratory aim was to examine whether variations in sleep-related HRV might be associated with next-day problem behaviors. Based on the hypothesis that increased sympathetic activity during sleep would predict the occurrence of problem behaviors on the following day, we compared HRV during nights preceding days with and without reported problem behaviors. However, no statistically significant differences were observed between the two conditions. Recent meta-analytic and theoretical work has emphasized that autonomic alterations in ASD are heterogeneous and not consistently observed across studies (17, 18). Thus, the null findings in this single-case dataset should not be interpreted as evidence of no relationship, but rather as reflecting this heterogeneity and the limited statistical power of the present design.

These results suggest that it may be difficult to directly predict next-day problem behaviors based solely on total sleep duration and HRV indices. HRV indices also showed considerable night-to-night fluctuation even on nights with similar caregiver-reported sleep duration, further limiting the ability to detect stable associations within this dataset. One possible explanation lies in the multifactorial nature of problem behaviors in children with ASD, which are known to serve four primary functions: access to tangibles, attention-seeking, escape from aversive stimuli, and automatic reinforcement (37). Given the strong interaction between these behaviors and environmental contingencies, HRV alone may not be sufficient as a predictive variable. To improve prediction accuracy, it may be necessary to obtain more detailed information about each day's problem behaviors. Although caregivers in this study reported whether problem behaviors occurred and described their nature, future studies should also consider collecting information on the intensity of such behaviors. This could help reveal patterns indicating, for example, that problem behaviors are more intense following poor sleep and less intense following better sleep. Goodwin et al. (38) demonstrated the use of actigraphy—a wrist-worn accelerometer—for quantifying problem behaviors in individuals with ASD. Actigraphy allows for the measurement of behavior that is difficult to capture through human observation, including frequency, duration, and temporal patterns. The application of such objective behavioral quantification methods in future studies may lead to more accurate and consistent prediction of problem behaviors. Future work may integrate garment-based HRV monitoring with wrist-worn actigraphy and standardized caregiver reports, aligning these modalities within shared temporal windows to better model the dynamic interactions between sleep-related autonomic activity, movement patterns, and observed behaviors.

To date, most studies on sleep in children with ASD have employed short-term polysomnographic assessments, while even longer-term studies have largely relied on caregiver-reported questionnaires or visual observation. Few, if any, longitudinal studies have examined sleep-related HRV specifically. Given that sleep patterns fluctuate from day to day—and even more so in children with ASD compared to their typically developing peers (39)—short-term measurements are unlikely to capture stable sleep profiles. To determine which types of sleep changes are associated with behavioral outcomes, long-term monitoring is essential. This study is, to our knowledge, the first to measure HRV during sleep over a one-month period in a child with ASD and to examine its relationship with next-day problem behaviors. Although the findings did not support the predictive utility of HRV in this context, this study represents an important first step in exploring physiological-behavioral associations in naturalistic settings. Longitudinal designs not only enable between-subject analyses but also facilitate the modeling of within-subject variability, which is particularly relevant for predicting individualized behavioral responses. In the future, sleep-related HRV may also be useful for predicting other socially significant responses, such as joint attention (40) and emotional regulation (41).

The present study also demonstrated the utility of garment-type wearable devices for real-time measurement of everyday sleep. While not as precise as polysomnography and limited in the range of physiological signals they can capture (42), wearable garments are well-suited for long-term sleep monitoring. From a feasibility standpoint, one of the two initially enrolled children was unable to tolerate the garment due to pronounced sensory hypersensitivity. This highlights the need for design considerations such as fabric texture, seam placement, and graded familiarization protocols in future wearable systems for ASD. Across the 32 planned nights, 25 (78%) yielded usable paired physiological and behavioral data, suggesting that this level of adherence may represent a realistic expectation for month-long home-based monitoring. In particular, the reduced noise during sleep enhances the reliability of HRV data collection. With the continued accumulation and analysis of high-quality physiological data during sleep, such measures may eventually be used to predict a variety of psychological and behavioral outcomes. Although garment-type wearables have been reported to be tolerable for children with ASD, including those with sensory sensitivities (28), the present study showed that in cases of extreme sensory hypersensitivity, sustained use of the device may be difficult. Thus, future studies should consider using sensory profiling tools [e.g., (43, 44)] to assess the likelihood that children will tolerate wearable devices for physiological monitoring.

A key limitation of the present study is that it involved only a single participant. To further examine the predictive value of HRV during sleep for problem behaviors, future studies should include participants who exhibit a broader range of behaviors in terms of intensity, frequency, and topography. It may also be beneficial to collect additional profile variables, such as cognitive and language development, adaptive behavior, and ASD symptom severity, to examine how the relationship between sleep-related HRV and behavior may vary across subgroups.

If behavioral prediction based on physiological and other quantitative indices becomes feasible, it may eventually enable caregivers and professionals to anticipate episodes of challenging behavior and implement preventive interventions (45–47). Such predictive systems may be especially beneficial for children who engage in frequent and severe behaviors, or for those with limited verbal repertoires who struggle to communicate internal states. In these cases, physiological signals may offer a critical early warning system to reduce the risk of problem behavior and enhance proactive support.

## Data Availability

The raw data supporting the conclusions of this article will be made available by the authors, without undue reservation.
